# Supplementary material for: Physiologically Based Pharmacokinetic Modeling of Extracellular Vesicles
Source: Biology (Basel). 2023 Aug 29;12(9):1178. doi: 10.3390/biology12091178 (PMC10525702; doi:10.3390/biology12091178)
Supplement: Supplementary file 1 [file biology-12-01178-s001.zip › biology-2527753-supplementary.pdf]

Supplementary Information

Physiologically Based Pharmacokinetic Modeling of Extracellular Vesicles

Prashant Kumar [1,*], Darshan Mehta [1] and John J. Bissler [2]

[1] Division of Biochemical Toxicology, National Center for Toxicological Research, United States Food and Drug Administration, Jefferson, AR 72079, USA; darshan.mehta@fda.hhs.gov

[2] Department of Pediatrics, Division of Pediatrics Nephrology, University of Tennessee Health Science Center, Memphis, TN 38103, USA; jbissler@uthsc.edu

* Correspondence: prashant.kumar@fda.hhs.gov

Tabe S1. List of completed or ongoing clinical trials related to Extracellular vesicles in various diseases condition.

No.	ClinicalTrials.gov Identifier	Name of trial	status	diseases	EV source	EV Purpose	Location
**Cancer**							
1	NCT04394572	Identification of New Diagnostic Protein Markers for Colorectal Cancer	Completed	Colorectal Cancer	Biological: Blood sample	Biomarker Diagnostic	Chu Reims Reims, France
2	NCT04939324	Molecular Profiling of Exosomes in Tumor-draining Vein of Early-staged Lung Cancer	Recruiting	Lung Cancer Exosomes Non-Small Cell Lung Cancer	Biological: Blood samples at 2 sites: peripheral vein and tumor-draining vein	Diagnostic	CHU de Limoges Limoges, France
3	NCT03608631	iExosomes in Treating Participants With Metastatic Pancreas Cancer With KrasG12D Mutation	Recruiting	• KRAS NP_004976.2:p.G12D<br>• Metastatic Pancreatic Adenocarcinoma<br>• Pancreatic Ductal Adenocarcinoma<br>• Stage IV Pancreatic Cancer AJCC v8	Drug: Mesenchymal Stromal Cells-derived Exosomes with KRAS G12D siRNA)	Therapeutics	M D Anderson Cancer Center Houston, Texas, United States
4	NCT03830619	Serum Exosomal Long	Completed	Lung Cancer (Diagnosis)	Blood plasma	Diagnostics	Union Hospital,

		Noncoding RNAs as Potential Biomarkers for Lung Cancer Diagnosis					Tongji Medical College, Huazhong University of Science and Technology Wuhan, Hubei, China
5	NCT02977468	Effects of MK-3475 (Pembrolizumab) on the Breast Tumor Microenvironment in Triple Negative Breast Cancer (Pembro/IORT)	Recruiting	Triple Negative Breast Cancer	• Drug: Merck 3475 Pembrolizumab<br>• Radiation: Intraoperative radiation therapy (IORT)	Therapeutics	Columbia University Irving Medical Center New York, New York, United States
**Coronavirus**							
1.	NCT05216562	Efficacy and Safety of EXOSOME-MSC Therapy to Reduce Hyper-inflammation In Moderate COVID-19 Patients	Recruiting Phase 2 Phase 3	SARS-CoV2 Infection	- Drug: Exosome-MSC Intravenous injection<br>- Drug: Placebo Intravenous Injection<br>- Drug: COVID-19 Standard Treatment	Therapeutics	- RSPAD Gatot Soebroto Jakarta, DKI Jakarta, Indonesia<br>- RSUP Dr. M. Jamil Padang, West Sumatra

							, Indonesia<br>- RSUP Dr. Sardjito Yogyakarta, Indonesia
2.	NCT04747574	Evaluation of the Safety of CD24-Exosomes in Patients With COVID-19 Infection	Recruiting Phase 1	SARS-CoV-2	Drug: EXO-CD24	Therapy	Tel Aviv Medical Center Tel Aviv, Israel
**3.**	NCT04491240	Evaluation of Safety and Efficiency of Method of Exosome Inhalation in SARS-CoV-2 Associated Pneumonia.	Completed Phase 1 Phase 2	- Covid19<br>- SARS-CoV-2 PNEUMONIA<br>- COVID-19	- Drug: EXO 1 inhalation<br>- Drug: EXO 2 inhalation<br>- Drug: Placebo inhalation	Therapy	Medical Centre Dinasty Samara, Russian Federation
**Cardiovascular Diseases**							
1.	NCT03034265	New Biomarkers and Difficult-to-treat Hypertension	Completed	Hypertension	Urinary exosomes	Diagnostics	Department of Nephrology and Hypertension, Inselspital, Bern University

							Hospital Bern, Switzerland
2.	NCT05370105	Extracellular Vesicles as Stroke Biomarkers	Recruiting	- Stroke<br>- Rehabilitation	Blood	Diagnostics	- IRCCS Don Gnocchi, Fondazione Don Carlo Gnocchi ONLUS Florence, Italy<br>- IRCCS S. Maria Nascente, Fondazione Don Carlo Gnocchi ONLUS Milano, Italy
3.	NCT03478410	Role of Exosomes Derived From Epicardial Fat in Atrial Fibrillation	Recruiting	Atrial Fibrillation	Epicardial fat derived exosomes	Pathophysiology analysis	Sheba medical center Ramat Gan, Israel
4.	NCT02931045	Antiplatelet Therapy Effect on	Completed	Myocardial Infarction	Platlets	Pathophysiology analysis	- Laboratory of

		Extracellular Vesicles in Acute Myocardial Infarction	Phase 4				Experimental Clinical Chemistry, Academic Medical Centre of the University of Amsterdam Amsterdam, Netherlands - 1st Chair and Department of Cardiology, Medical University of Warsaw Warsaw, Poland

Tabe S2. A comparison between GastroPlus, Simcyp, PK-Sim and Berkely Madonna.

Feature	GastroPlus	Simcyp	PK-Sim	Berkeley Madonna
Developer	Simulations Plus	Certara	OPEN SYSTEMS PHARMACOLOGY	University of California, Berkeley
Purpose	Predictive PK modeling	PBPK modeling	PBPK modeling	Dynamic systems modeling
Application	Oral, IV, dermal, inhaled dosing	Diverse routes of administration	Diverse routes of administration	Dynamic systems modeling
Mechanistic Modeling	Detailed mechanistic modeling	Mechanistic and empirical models	Mechanistic and empirical models	Ordinary Differential Equations
Integration	In vitro, in vivo data integration	Incorporates clinical data	Integrates clinical data	Mathematical modeling
Specialization	Biopharmaceutics, formulation	Drug-drug interactions	Pediatrics, special populations	General mathematical modeling
Populations	Adult, pediatric, special groups	Wide range of populations	Diverse populations	General population
User-Friendly Interface	Graphical interface for modeling	User-friendly interface	Intuitive graphical interface	Graphical interface for modeling
Validation and Adoption	Widely adopted and validated	Industry standard	Widely accepted	Used in academia and research
Licensing	Commercial software	Commercial software	Commercial software	Commercial software
